# AI-assisted and Big-Unit teaching enhance speed-skating performance through psychological mechanisms in adolescents: evidence from a three-arm intervention study

**DOI:** 10.3389/fpsyg.2026.1750654

**Published:** 2026-02-02

**Authors:** Yongheng Zhao, Yunbo Wang, Limeng Liu, Chi Ma, Zhongtang Li

**Affiliations:** 1 School of Wushu, Henan University, Kaifeng, China; 2 Graduate School, Kyungil University, Gyeongsan-si, Republic of Korea; 3 School of Physical Education and Health Science, Mudanjiang Normal University, Mudanjiang, China; 4 Graduate School, Harbin Sport University, Harbin, China; 5 School of Physical Education, Jiangsu Second Normal University, Nanjing, China

**Keywords:** adolescent development, AI-assisted teaching, Big-Unit teaching, learning motivation, physical education, psychological resilience, self-efficacy, speed skating

## Abstract

**Background:**

Innovative instructional approaches are increasingly advocated in physical education to enhance both motor skill development and psychological adaptation. However, few studies have directly compared micro-level (AI-assisted) and macro-level (Big-Unit) teaching models, or examined the psychological mechanisms underlying performance improvements in adolescent winter-sport environments.

**Methods:**

A three-arm, quasi-experimental longitudinal study was conducted with 129 first-year middle school students (AI-assisted: *n* = 42; Big-Unit: *n* = 43; Conventional: *n* = 44). Participants completed an 8-week speed-skating intervention consisting of 24 on-ice lessons. Learning motivation, self-efficacy, psychological resilience, and related psychological constructs were assessed at baseline (T1), mid-intervention (T2), and post-intervention (T3). Skating performance was evaluated using electronic 500-m timing. Linear mixed-effects models, ANCOVA, and structural equation modeling were applied to assess Group × Time interactions and mediation pathways.

**Results:**

Both AI-assisted and Big-Unit teaching produced significantly larger improvements in 500-m performance than conventional instruction (AI: −5.59 s; Big-Unit: −7.60 s; Conventional: −1.80 s; all *p* < 0.001). All 13 psychological outcomes showed strong Group × Time interactions favoring the innovative groups [all χ^2^_(4)_ > 137.28, *q* < 0.001]. ANCOVA confirmed substantial adjusted Group effects for changes in learning motivation, self-efficacy, psychological resilience, and anxiety/stress (partial η^2^ = 0.650–0.927). Mediation analyses identified a statistical suppression pattern, in which increases in learning motivation and self-efficacy served as significant indirect pathways linking innovative instruction to performance gains. However, the direct technical impact remained the dominant driver.

**Conclusion:**

AI-assisted and Big-Unit teaching substantially enhance both technical performance and psychological functioning in adolescent speed skating. Statistical mediation models support learning motivation as a plausible mechanism linking teaching mode to performance, with self-efficacy providing additional support. These findings highlight the complementary potential of technology-enhanced and mastery-oriented pedagogies to modernize physical education through both direct technical renovation and indirect psychological adaptation.

## 1 Introduction

Physical education (PE) plays a critical role in supporting adolescents' physical, motivational, and emotional development. Yet global surveillance indicates that over 80% of adolescents fail to meet recommended activity levels, accompanied by declining motivation and rising academic stress (Holmes et al., 2019). These patterns underscore the pressing need for pedagogical innovations that not only enhance skill performance but also foster adaptive psychological processes—particularly motivation, self-efficacy, and resilience—that sustain engagement in the face of challenges.

Two promising instructional approaches have emerged in recent years: AI-assisted teaching and Big-Unit teaching (a large-unit curriculum model). AI-assisted instruction integrates motion-recognition technologies and real-time corrective feedback to refine movement execution (Ryan and Deci, 2000). According to Self-Determination Theory (SDT), competence-supportive and autonomy-supportive feedback promotes intrinsic motivation by satisfying basic psychological needs. Social Cognitive Theory further proposes that repeated successful corrections strengthen self-efficacy, a key predictor of persistence in complex motor tasks (Moritz et al., 2000). However, ethical and pedagogical considerations must guide the integration of AI: excessive reliance may undermine autonomy, create cognitive overload, or introduce algorithmic bias (Guadagnoli and Lee, 2004; Holmes et al., 2019). When mediated by teachers—who regulate pacing, interpret feedback, and ensure fairness—AI can enhance rather than replace students' self-regulated learning, contributing to a psychologically supportive learning climate.

Big-Unit teaching represents a complementary innovation at the curriculum level. Rather than delivering fragmented lessons, Big-Unit instruction organizes learning into extended, coherent units with scaffolded task progressions and explicit mastery criteria. This structure aligns with mastery motivational climate research, grounded in achievement goal theory (Ames, Duda, Nicholls; Ames, 1992; Roberts and Treasure, 2012), which emphasizes task involvement, perceived competence, and personal improvement. By normalizing temporary errors, encouraging effort, and providing repeated opportunities for mastery, Big-Unit teaching environments foster intrinsic motivation and adaptive coping strategies. These features are also consistent with the Challenge Point Framework and ecological dynamics perspectives, which highlight graded difficulty, feedback dependency, and perception–action coupling in motor learning. Together, such instructional features are theorized to strengthen psychological resilience—conceptualized as positive adaptation under challenge—and be modifiable through structured exposure to progressively demanding tasks and supportive climates in youth sports (Masten, 2001; Guadagnoli and Lee, 2004; Davids et al., 2008; Arnetz et al., 2009; Sarkar and Fletcher, 2014).

Speed skating offers a particularly relevant context for examining these mechanisms. The 500-m event is a cyclic, high-precision skill characterized by strong error sensitivity, substantial feedback dependence, and complex perceptual–motor coupling. Ice-based movement elevates postural threat and fear of falling, increasing anxiety and requiring robust motivational and self-efficacy resources to maintain performance (De Souza et al., 2015; Ellmers et al., 2024). Given the growing integration of winter sports into PE curricula, evidence-based instructional models for supporting both skill acquisition and psychological readiness in this high-demand environment are urgently needed.

Although prior studies show that AI feedback and mastery-oriented curricula can enhance student learning, three significant gaps remain. First, few investigations have directly tested the psychological mechanisms—particularly changes in motivation, self-efficacy, and resilience—that underlie the performance gains from innovative pedagogies in PE. Second, AI-assisted instruction (a micro-level feedback innovation) and Big-Unit teaching (a macro-level structural innovation) represent distinct theoretical pathways. A head-to-head comparison is necessary to determine whether they activate overlapping or differentiated psychological processes, thereby informing future integrated teaching models. Third, no existing study has simultaneously contrasted these pedagogies, modeled psychological mediators, and assessed performance outcomes in an authentic adolescent winter-sport PE environment.

The present study addressed these gaps through an 8-week, three-arm intervention that compared AI-assisted teaching, Big-Unit teaching, and conventional instruction in middle school speed skating. Guided by SDT, Social Cognitive Theory, mastery climate research, and contemporary resilience models, we examined three psychological constructs central to engagement in challenging motor learning contexts. Learning motivation was conceptualized as the primary mediator linking teaching mode to performance improvement. Self-efficacy was treated as a theoretically relevant but secondary pathway. Psychological resilience—conceived as a trainable constellation of coping skills, adaptive self-regulation, and challenge readiness—was examined as a key outcome expected to improve under supportive instructional climates.

This study pursued three aims (Masten, 2001; Sarkar and Fletcher, 2014; Godor, 2025):

- to compare performance improvements across AI-assisted, Big-Unit, and conventional instruction;
- to examine differential gains in learning motivation, self-efficacy, and resilience;
- to test whether motivational and efficacy gains mediate the performance advantages of innovative instruction.

These aims yielded the following hypotheses:

H1. AI-assisted and Big-Unit instruction will produce greater improvements in 500-m skating performance than conventional teaching.
H2. Both innovative approaches are expected to yield larger gains in learning motivation, self-efficacy, and resilience.
H3a. Increases in learning motivation will mediate the relationship between teaching mode and performance improvement.
H3b. Increases in self-efficacy will provide a weaker but parallel mediating pattern.

**Figure 1:**
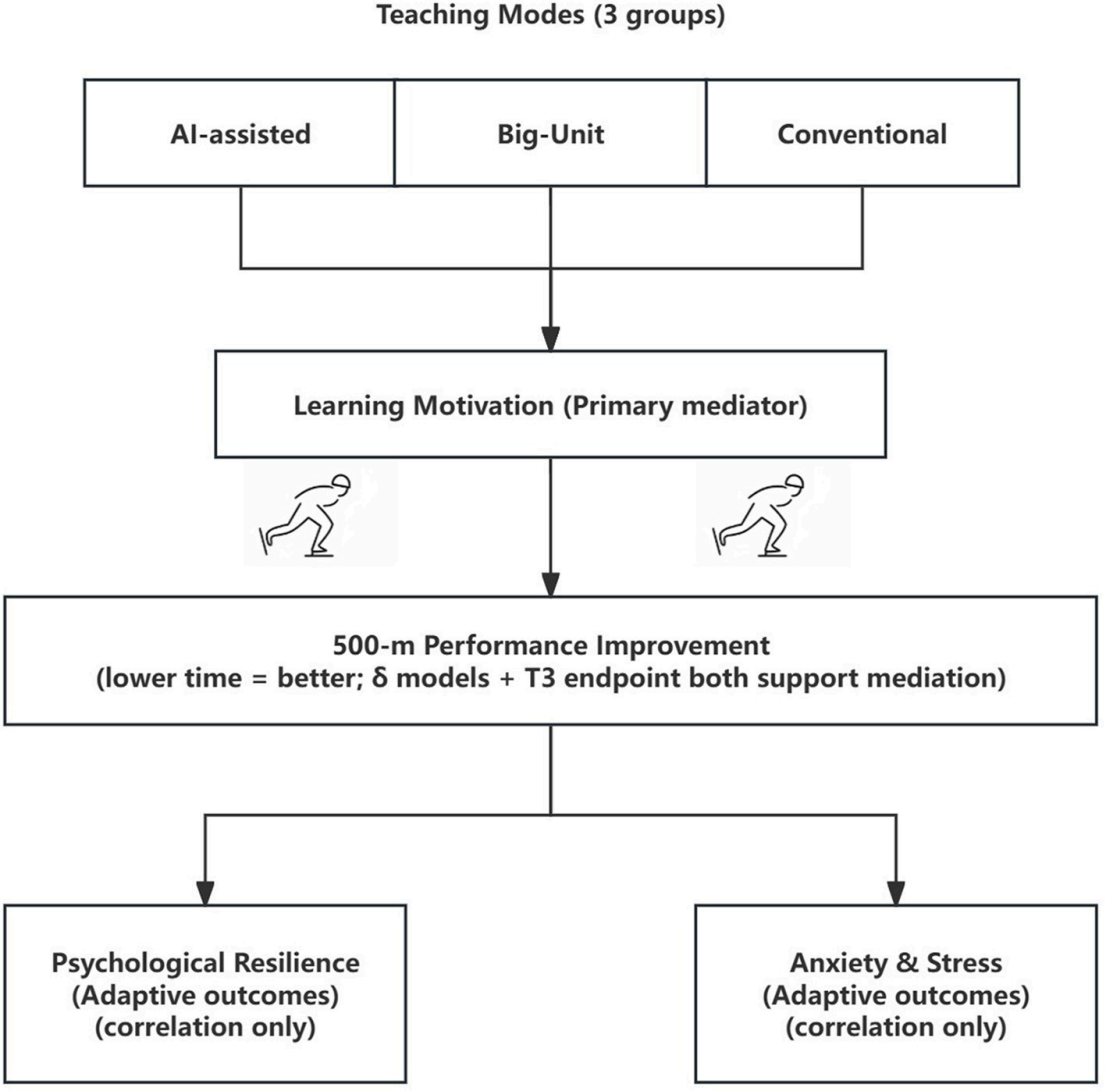
Baseline characteristics of the participants by instructional group (AI-assisted, Big-Unit, Conventional).

A conceptual model of the hypothesized mechanisms is presented in Figure 1. To our knowledge, this is the first study to directly compare AI-assisted and Big-Unit instruction, examine psychological mediators, and evaluate performance outcomes in an authentic adolescent winter-sport PE context, providing novel theoretical and practical contributions for technology-enhanced and mastery-oriented pedagogy in PE.

## 2 Methods

### 2.1 Study design and implementation context

This study employed a three-arm, quasi-experimental longitudinal design with repeated assessments at three time points (T1, T2, T3; Cheon et al., 2012). The intervention was implemented during the winter term of 2024 at a Korean-ethnicity middle school in Mudanjiang, Heilongjiang Province, China. In this region, speed skating is a compulsory component of the primary school physical education curriculum; thus, students typically enter lower secondary school with 1–2 years of basic skating experience (e.g., forward stride, blade-angle control, basic braking, and safety; Ning et al., 2022). This “baseline familiarity but non-professional skill level” profile provides a developmentally appropriate context for evaluating instructional effects in a technically demanding winter sport.

To ensure safety and standardize basic technique, all participants completed a 2-week dryland preparatory phase before the formal intervention. These sessions focused on balance training, posture, and weight-shift control, stride simulation, rhythm drills, and safety instructions (Zadic et al., 2025). Because this phase was intended only to equalize baseline readiness, it was not included in the experimental period or analyses.

The formal on-ice intervention commenced on 18 November 2024, coinciding with the period when outdoor school ice tracks in Mudanjiang typically achieve stable freezing conditions. The intervention lasted 8 weeks, with each group receiving three 45-min sessions per week (24 sessions in total) in accordance with the school's PE timetable. To enhance ecological validity, classes were conducted primarily on the school's 500-m outdoor artificial ice track constructed by layered flooding. When outdoor temperatures fluctuated or ice conditions were compromised by snow, melt events, or surface instability, sessions were moved to a partnered indoor artificial ice facility to maintain safety and instructional continuity (McLeman et al., 2024).

Assessments were scheduled as follows:

- T1 (baseline): 1 week before the intervention;
- T2 (mid-test): at the end of Week 4;
- T3 (post-test): within 1 week after the intervention.

**Figure 2:**
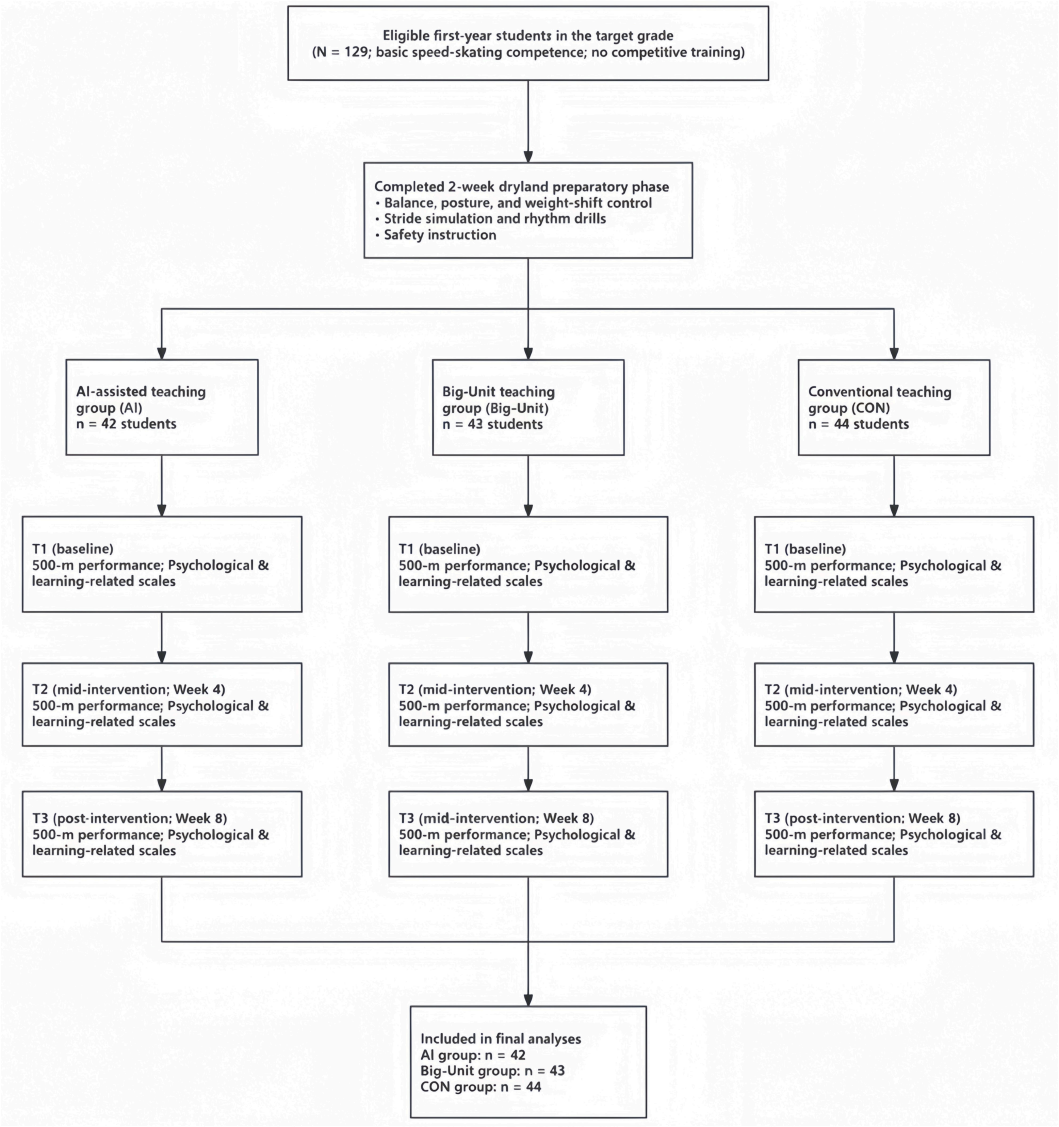
Study design and participant flow for the three instructional groups (AI-assisted, Big-Unit, and Conventional).

This design allowed the examination of both immediate and cumulative changes in skating performance and psychological variables across instructional conditions. The overall study design and participant flow are summarized in Figure 2.

### 2.2 Participants and group allocation

Participants were first-year middle school students at the participating school. A total of 129 students (76 boys, 53 girls) with complete data at all three time points were included in the analytic sample (AI-assisted group: *n* = 42; Big-Unit group: *n* = 43; Conventional group: *n* = 44). The mean baseline age was 14.5 ± 0.5 years. All students had previously completed the regional compulsory primary school speed-skating curriculum and were able to skate independently, with basic technical control and ice safety awareness.

Because the school's class structure could not be reorganized, an intact-class allocation strategy was adopted, a common approach in educational intervention research (Cheon et al., 2012). Before allocation, the three classes were verified to be similar in terms of age distribution, sex ratio, prior skating experience, weekly PE schedule, and overall academic environment. Given the quasi-experimental, intact-class design, teaching mode was partially confounded with class-level context (e.g., peer dynamics or specific class climates). To mitigate potential selection bias and bolster internal validity, our statistical analyses prioritized within-student longitudinal changes and utilized baseline-adjusted ANCOVA and mediation models. The three natural classes were assigned to:

- AI-assisted teaching group (AI group)
- Big-Unit teaching group (BU group)
- Conventional teaching group (CON group)

This quasi-experimental, intact-class design preserves ecological validity and prevents contamination between groups (e.g., by avoiding the sharing of teaching materials within classes). At the same time, it entails partial confounding between teaching mode and class-level context, a limitation acknowledged in the Discussion. Before allocation, the three classes were verified to be similar in terms of age distribution, sex ratio, prior skating experience, weekly PE schedule, and overall academic environment.

Inclusion criteria were:

- enrolment in the target grade at the participating school;
- basic speed-skating competence (able to complete one lap independently);
- no cardiovascular, neuromuscular, or orthopedic conditions limiting participation in PE;
- no history of competitive or specialized speed-skating training;
- ability to participate in on-ice classes without external assistance;
- provision of written informed consent from parents or legal guardians.

Students were excluded if they: (a) missed more than two consecutive weeks of the intervention; (b) sustained injuries or illnesses preventing continued participation; or (c) had incomplete data at any of the three assessment points. All procedures conformed to the Declaration of Helsinki and were approved by the Ethics Committee of the School of Physical Education, Mudanjiang Normal University (approval number: MDNU-REC-PE-2024-056; Bolin, 2014). For mediation analyses and some secondary models, teaching mode was recoded as a binary contrast: Innovative instruction (AI-assisted + Big-Unit = 1) vs. Conventional instruction (0).

### 2.3 Intervention protocol

The intervention spanned 8 weeks, with three 45-min PE lessons per week for each group (24 sessions total). All groups received identical scheduled exposure (45 min/session, 3 sessions/week); the interventions differed in instructional strategy rather than total practice time, ensuring time-on-task equivalence across conditions. To minimize instructor-related variability, the same experienced PE teacher, trained in both AI-assisted and Big-Unit instructional methods, delivered all lessons across the three conditions (Mosston and Ashworth, 2002). All sessions were conducted in accordance with standard winter-sport safety protocols (Wang et al., 2021).

#### 2.3.1 AI-assisted teaching group

The AI group received instruction supported by a video-based motion recognition and performance-analysis system (e.g., SpeedSkate-AI Analysis System v1.0) capable of estimating stride frequency, stride length, joint angle patterns, center-of-mass movement, and skating trajectories. The system utilized two fixed cameras (1080p, 30 fps) placed rink-side at approximately 5–8 m from the track. To ensure technical reliability, the system was field-tested and calibrated prior to the intervention to compensate for lens distortion and optimize tracking accuracy under varying rink lighting and surface reflections. During practice, each student completed repeated 500-m or partial-distance trials. Feedback was delivered via a station-based rotation at the rink-side station, ensuring each student received two scheduled feedback episodes per lesson, with each episode lasting approximately 30 s. After each trial, the system generated immediate visual feedback, including (Wulf et al., 2010):

- key technical deviations from the target model (e.g., excessive trunk flexion, inadequate knee flexion, asymmetrical push-off);
- stride frequency–speed relationships;
- trajectory stability curves;
- radar-chart summaries of technical consistency across laps.

The teacher interpreted these outputs in a competence-supportive manner, emphasizing specific, actionable corrections rather than evaluative judgments, consistent with Self-Determination Theory principles of autonomy and competence support (Ryan and Deci, 2000). To ensure standardized delivery, the teacher completed an 8-h workshop and a pilot session focused on system operation and translating kinematic data into non-judgmental feedback. For students exhibiting cautious movement patterns or reduced confidence, feedback was framed to emphasize incremental progress and actionable adjustments. The system operated without storing personal identifiers, and only anonymised performance data (e.g., coded ID, kinematic variables) were processed. Data collection followed principles of data minimization and privacy protection: only variables necessary for instructional feedback and research analyses were retained (Holmes et al., 2019).

#### 2.3.2 Big-Unit teaching group

The Big-Unit group followed a structured curriculum organized into three coherent instructional units grounded in constructivist and mastery-learning frameworks (Block and Burns, 1976): To operationalize the mastery-learning approach, the progress of each student was monitored using a standardized technical rubric:

- **Unit 1 (Weeks 1–2):**

Focus on fundamental posture, balance, and braking. Instruction involved task decomposition (e.g., static balance, glide-and-freeze, single-leg stance), peer observation, and guided feedback to reduce cognitive load and strengthen foundational control. Mastery for this unit was defined as: (a) maintaining a single-leg glide balance for ≥5 s on either blade, and (b) executing a controlled braking maneuver within a 2-m designated zone.

- **Unit 2 (Weeks 3–5):**

Emphasis on rhythm control, stride acceleration, and multi-pace transitions. Learning tasks were linked into progressive “task chains” (e.g., slow–medium–fast laps, pace changes at pre-set markers), helping students construct a coherent representation of speed management and energy distribution. Mastery criteria included: (a) completing three consecutive paced laps within a time variance of ±2 s, verified via electronic timing, and (b) demonstrating rhythmic arm-swing coordination consistent with stride frequency.

- **Unit 3 (Weeks 6–8):**

Integration of full 500-m performance, including pacing strategies, cornering technique, error correction, and reflective practice (e.g., self-evaluation after timed trials). Mastery was assessed based on trajectory stability during high-speed cornering (no visible outward drifting) and the accuracy of post-trial self-reflection in identifying specific technical errors.

Mastery checks were conducted by the teacher at the end of each unit using standardized checklists. Importantly, if a student did not meet the predefined criteria, they were provided with individualized 5-min remedial practice sessions focusing on their specific skill deficits within the same lesson. These sessions ensured all students attained foundational proficiency before advancing to subsequent units, thereby promoting sustained engagement, tolerance of temporary failure, and the gradual development of psychological resilience (Sarkar and Fletcher, 2014).

#### 2.3.3 Conventional teaching group

The Conventional teaching group followed the standard regional PE curriculum for speed skating. Instruction relied primarily on teacher demonstration, whole-group practice, and repetitive full-lap drills, without AI-based feedback or structured Big-Unit task progression. Technical guidance was provided through general verbal correction rather than individualized or data-informed feedback (Latinjak et al., 2019). Lesson flow followed the traditional 45-min PE format, with limited task decomposition and minimal scaffolding across sessions.

Students typically performed:

- whole-group skating drills,
- repeated 500-m or partial-lap trials,
- teacher-led correction based on visual observation,
- basic safety reminders (e.g., maintaining distance, controlled pacing).

Compared with the AI-assisted and Big-Unit groups, the Conventional group received neither real-time kinematic feedback nor mastery-oriented unit structuring. Thus, this condition functioned as a pragmatic active control reflecting typical school-based PE practice in the region.

#### 2.3.4 Intervention fidelity

To maintain high implementation standards and ensure protocol adherence across the 8-week study, intervention fidelity was systematically monitored. A non-teaching research assistant, pre-trained in the study protocols, observed approximately 25% of the total lessons (six sessions per group) using a standardized fidelity checklist. The checklist documented: (i) the consistent delivery of AI feedback episodes (frequency and 30-s duration); (ii) the systematic implementation of Big-Unit task sequences and unit-end mastery checks; and (iii) the absence of experimental feedback in the Conventional group. Furthermore, the assistant attended weekly debriefing sessions with the teaching staff to review observation records and address any potential protocol drift. Across all monitored sessions, core intervention components were delivered as planned with high adherence (no major deviations recorded), ensuring that the observed effects were attributable to the intended instructional strategies rather than extraneous variability.

### 2.4 Measures

All outcome measures were administered at T1 (baseline), T2 (mid-intervention), and T3 (post-intervention) during regular PE periods, under the supervision of the research team and school staff.

#### 2.4.1 Skating performance (500-m time)

The primary outcome was the 500-m speed skating performance time (SkillTime). Students completed two timed 500-m trials on the school's standard track at each assessment point, with at least 5 min of active recovery between trials. Times were recorded using a dual-beam electronic timing system. For analysis, the faster of the two times was used. Test–retest reliability for the 500-m time in a subsample of students yielded an intraclass correlation coefficient (ICC) of 0.92, indicating excellent measurement stability (Latinjak et al., 2019).

#### 2.4.2 Psychological and learning-related constructs

A battery of validated Chinese questionnaires was administered to capture students' motivational, competence-related, and emotional adaptation to the intervention. All scales used a five-point Likert format (1 = strongly disagree to 5 = strongly agree), with higher scores indicating higher levels of the respective construct. The following constructs were included:

- **Learning motivation (main scale):** assessed using the Chinese Physical Education Learning Motivation Scale (PELM-S; 12 items), capturing interest, intrinsic regulation, and perceived competence in PE. Previous studies in Chinese adolescents have reported good internal consistency (Cronbach's α ≈ 0.85–0.90) and construct validity (Zhang et al., 2024).
- **Exercise interest and pleasure:** subscales assessing enjoyment of speed-skating lessons and interest in continued participation in skating-related activities (Zhao et al., 2025).
- **Autonomous learning ability:** items assessing students' perceived ability to self-direct practice, set personal goals, and evaluate their own progress in PE.
- **Core competence and cooperation competence:** scales indexing key PE-related core qualities, including tactical understanding, problem-solving, teamwork, and cooperation in practice settings.
- **Self-efficacy (main PE-related scale):** items reflecting confidence in mastering new skating skills, coping with complex tasks, and maintaining effort under fatigue.
- **Anxiety and stress in PE:** items measuring perceived nervousness, performance-related worry, and stress during skating classes and assessments.
- **Emotional regulation and self-control:** scales assessing the ability to manage negative affect, maintain focus, and inhibit impulsive behavior during training.
- **Psychological resilience:** assessed via a 10-item Athletic Resilience Scale adapted for physical activity contexts, capturing recovery from setbacks, perseverance, and optimistic coping; prior work in Chinese youth has shown good reliability (Cronbach's α ≈ 0.85; Hu and Gan, 2008).

Two supplementary composite indices—supplementary learning motivation and supplementary self-efficacy—were derived from additional items focusing on speed-skating–specific attitudes and confidence. In total, 13 psychological and learning-related outcomes (11 primary scales plus 2 supplementary composites) were included in the longitudinal analyses. While primary scales were used to establish longitudinal trends, the supplementary scales were specifically utilized for the mediation and ANCOVA models. These scales were linguistically tailored to the speed-skating context (e.g., addressing ice-surface anxiety and confidence in pacing strategies), providing higher sensitivity to the situational psychological shifts induced by the innovative interventions than general-purpose PE instruments. This context-to-construct alignment was intended to maximize the ecological validity of the mechanistic findings in this specialized winter-sport environment.

#### 2.4.3 Anthropometrics and demographics

Height and weight were measured by trained PE staff using standardized procedures, and body mass index (BMI) was calculated as weight (kg) divided by height (m^2^). BMI categories (normal weight, obese) were defined according to national student fitness standards. Students also reported their age, gender, and the approximate duration of their prior skating experience (in years).

### 2.5 Statistical analysis

All analyses were performed in R (R Foundation for Statistical Computing, Vienna, Austria; R Core Team, 2024). Two-sided *p*-values < 0.05 were considered statistically significant unless otherwise specified. Continuous variables are reported as mean ± SD, and categorical variables are reported as frequencies and percentages. Analyses were conducted on the completer dataset (*n* = 129), and no imputation was performed for missing repeated-measures data.

#### 2.5.1 Baseline characteristics

Between-group comparability at T1 was evaluated before outcome analyses. Sex and BMI-category distributions were compared using chi-square tests. Continuous variables—including age, height, weight, BMI, baseline 500-m performance, and all psychological scales—were compared using one-way ANOVA with Group as the between-subject factor. For each variable, F-statistics, *p*-values, and partial eta squared were reported (Lakens, 2013).

Variables exhibiting significant baseline differences (learning motivation and psychological resilience) were included as covariates in subsequent ANCOVA and mediation models to account for potential baseline-related bias. Given the intact-class setting, baseline differences in motivation and resilience may reflect pre-existing class climate, peer dynamics, or teacher–student interaction patterns prior to the study. Therefore, baseline values of these constructs were statistically controlled in ANCOVA and mediation models, and results were interpreted with caution regarding selection effects.

#### 2.5.2 Primary analysis: longitudinal mixed-effects models for 500-m performance

To evaluate time-varying intervention effects on 500-m performance, data were first reshaped into long format with Time treated as a categorical factor (T1, T2, T3). Linear mixed-effects models (LMMs) with a random intercept for participant were then fitted to estimate the fixed effects of Group, Time, and their interaction (Group × Time; Bates et al., 2015). Age, gender, and BMI were included as covariates.

Model parameters were estimated using maximum likelihood to enable likelihood-ratio tests (LRTs) for nested model comparisons (Bates et al., 2015). Specifically, LRTs were used to compare:

1. models with vs. without Time (overall temporal change across T1–T3);
2. models with vs. without the Group × Time interaction (differential change across groups); and
3. models with vs. without Group (overall group differences across instructional conditions).

Descriptive means and standard deviations for each Group × Time cell were reported to support interpretation of the longitudinal patterns. Performance improvement over the intervention was additionally summarized using simple change scores (ΔSkillTime = T3 – T1), with negative values indicating faster performance (better). Within-group repeated-measures effect sizes (Cohen's d and 95% confidence intervals) were computed using the *effectsize* package (Ben-Shachar et al., 2020).

#### 2.5.3 Longitudinal mixed-effects models for psychological and learning outcomes

For each of the 13 psychological and learning-related variables, linear mixed-effects models (LMMs) with random participant intercepts were fitted, with Group, Time, and their interaction (Group × Time) specified as fixed effects (Bates et al., 2015). To preserve the interpretability of raw developmental trajectories, no covariates were included in these primary psychological models.

For each outcome, a main-effects model (Group + Time) was compared with a complete interaction model (Group × Time) using likelihood-ratio tests (LRTs). To control for multiple testing across the family of 13 outcomes (11 primary scales and 2 supplementary scales), the Benjamini–Hochberg false discovery rate (FDR) procedure was applied (Benjamini and Hochberg, 1995). Both unadjusted *p*-values and FDR-adjusted q-values were reported. Covariate-adjusted specifications (age, gender, BMI) were examined as sensitivity analyses and are described in Section 2.5.6.

#### 2.5.4 Secondary analysis: ANCOVA on baseline-adjusted change scores

To complement the longitudinal mixed-effects models and obtain baseline-adjusted effect sizes, ANCOVAs were conducted on change scores (T3 – T1) for five outcomes: 500-m performance, supplementary learning motivation, supplementary self-efficacy, psychological resilience, and anxiety & stress. For each ANCOVA, Group served as the main predictor, and the model adjusted for the baseline outcome value (T1) and age, gender, and BMI.

Type III sums of squares were used to evaluate the adjusted Group effect, and partial eta squared (η^2^*p*) was reported as the primary effect-size index (Cohen, 2013).

#### 2.5.5 Associations and mediation analyses

Given the quasi-experimental design, mediation analyses were conducted to test statistically plausible pathways rather than causal mechanisms. Performance improvement was defined as the reduction in 500-m time during the intervention (Improvement = –ΔSkillTime), with larger values indicating greater improvement. Pearson correlations were computed between improvement scores and four psychological change scores (learning motivation, self-efficacy, psychological resilience, anxiety & stress).

To examine hypothesized psychological pathways, two single-mediator structural equation models (SEMs) were estimated using the *lavaan* package (Rosseel, 2012):

- teaching mode → change in learning motivation → performance improvement,
- teaching mode → change in self-efficacy → performance improvement.

Teaching mode was coded as Innovative (AI + Big-Unit = 1) vs. Conventional (0). Both the mediator and outcome regressions controlled for the corresponding baseline mediator (T1) and baseline performance, along with age, gender, and BMI. Indirect effects were estimated using non-parametric bootstrapping (1,000 resamples); mediation was considered present when the 95% confidence interval for the indirect impact excluded zero (Bolin, 2014).

#### 2.5.6 Sensitivity analyses

Several predefined sensitivity analyses were conducted to assess robustness:

1. **Alternative longitudinal framework**
  Traditional repeated-measures ANOVAs (Group × Time) were fitted for key outcomes and compared qualitatively with LMM results (Gueorguieva and Krystal, 2004).
2. **Covariate adjustment strategies**
  For performance and selected psychological outcomes, three model specifications were compared: unadjusted, age-adjusted, and fully adjusted (age, gender, BMI). Group × Time conclusions remained consistent.
3. **Outlier removal**
  Participants with extreme change scores (|*z*| > 3) were excluded, and core analyses were repeated. Patterns of significance and effect sizes remained stable.
4. **Multiple-comparison control**
  All 13 psychological outcomes remained significant after FDR correction (Benjamini and Hochberg, 1995).
5. **Alternative operationalization of mediation**
  Mediation models using T3 outcomes with baseline values as covariates were fitted, confirming the robustness of learning-motivation mediation and the directionally consistent pattern for self-efficacy (Bolin, 2014).

Across all sensitivity tests, the magnitude and statistical significance of the primary findings remained unchanged, supporting the robustness of the intervention effects.

## 3 Results

### 3.1 Baseline characteristics

Baseline characteristics for the three instructional groups are summarized in Table 1. A total of 129 students completed all three assessments (AI = 42; Big-Unit = 43; Conventional = 44). Sex distribution did not differ significantly across the groups [χ^2^_(2)_ = 0.79, *p* = 0.675]. No significant between-group differences were observed in age, height, weight, or BMI (all *p* > 0.10). Baseline 500-m skating performance was also comparable across groups [*F*_(2, 126)_ = 1.09, *p* = 0.341]. BMI category included “Normal” and “Obese,” with no participants classified as “Overweight”; a corrected chi-square test after removing the empty “Overweight” level confirmed no group differences among participants with standard or obese BMI [*n* = 120; χ^2^_(2)_ = 0.05, *p* = 0.974].

**Table 1:** Baseline characteristics of the participants by instructional group (AI-assisted, Big-Unit, conventional).

**Variable**	**AI-assisted (*n* = 42)**	**Big-Unit (*n* = 43)**	**Conventional (*n* = 44)**	**Test statistic**	***p*-value**
Sex, *n* (%)	Male 26 (61.9%) Female 16 (38.1%)	Male 23 (53.5%) Female 20 (46.5%)	Male 27 (61.4%) Female 17 (38.6%)	χ^2^_(2)_ = 0.79	0.675
Age (years)	14.4 ± 0.49	14.6 ± 0.50	14.5 ± 0.50	*F*_(2, 126)_ = 2.31	0.104
Height (cm)	163.0 ± 8.7	168.0 ± 9.2	165.0 ± 9.6	*F*_(2, 126)_ = 2.23	0.112
Weight (kg)	65.8 ± 13.0	68.7 ± 13.6	68.0 ± 13.0	*F*_(2, 126)_ = 0.57	0.567
BMI (kg/m^2^)	25.7 ± 3.4	24.8 ± 3.2	24.9 ± 3.3	*F*_(2, 126)_ = 0.14	0.868
BMI category, *n* (%)^†^	Normal 18/Obese 22	Normal 18/Obese 20	Normal 19/Obese 23	χ^2^_(2)_ = 0.05	0.974
500-m time (s)	59.1 ± 6.78	59.9 ± 6.47	61.1 ± 5.58	*F*_(2, 126)_ = 1.09	0.341
Learning motivation (T1)	3.02 ± 0.45	3.34 ± 0.41	3.13 ± 0.44	*F*_(2, 126)_ = 36.18	< 0.001
Self-efficacy (T1)	3.20 ± 0.48	3.03 ± 0.46	3.07 ± 0.47	*F*_(2, 126)_ = 1.48	0.231
Psychological resilience (T1)	3.26 ± 0.43	3.21 ± 0.45	3.33 ± 0.42	*F*_(2, 126)_ = 38.97	< 0.001

†BMI category calculated for n = 120 participants. Nine Underweight cases were excluded from this categorical analysis to ensure statistical robustness, and the Overweight category contained zero cases.

For psychological variables, baseline scores were similar across groups for most measures. However, significant group differences were observed for learning motivation [supplementary scale; *F*_(2, 126)_ = 36.18, *p* < 0.001] and psychological resilience [main scale; *F*_(2, 126)_ = 38.97, *p* < 0.001]. These variables were therefore statistically controlled as covariates in subsequent ANCOVA and mediation analyses. All other psychological constructs, including self-efficacy [supplementary scale; *F*_(2, 126)_ = 1.48, *p* = 0.231], were comparable at baseline.

Overall, demographic, anthropometric, and performance characteristics were broadly comparable at baseline, with only two psychological constructs (learning motivation and psychological resilience) requiring statistical adjustment in later models.

### 3.2 500-m Speed-skating performance

Across the full sample (*n* = 129), 500-m speed-skating performance improved over time in all three groups, with clearly larger gains in the innovative teaching conditions (Table 2). At baseline (T1), mean 500-m times were comparable across groups (AI: 59.1 ± 6.78 s; Big-Unit: 59.9 ± 6.47 s; Conventional: 61.1 ± 5.58 s), consistent with the non-significant Group effect on T1 performance [*F*_(2, 126)_ = 1.09, *p* = 0.34].

**Table 2:** 500-m speed-skating performance across T1–T3.

**Group**	**T1 Mean ±SD (s)**	**T2 Mean ±SD (s)**	**T3 Mean ±SD (s)**	**Δ(T3 – T1) Mean ±SD (s)**	**Cohen's *d* (abs)**
AI-assisted	59.1 ± 6.78	56.3 ± 6.36	53.5 ± 6.04	−5.59 ± 1.10	5.10
Big-Unit	59.9 ± 6.47	55.9 ± 6.37	52.3 ± 6.00	−7.60 ± 1.86	4.09
Conventional	61.1 ± 5.58	60.2 ± 5.52	59.3 ± 5.56	−1.80 ± 0.82	2.20

Δ(T3 – T1) < 0 indicates faster performance (better). Cohen's d is the absolute value of the repeated-measures effect size (T1–T3).

**Figure 3:**
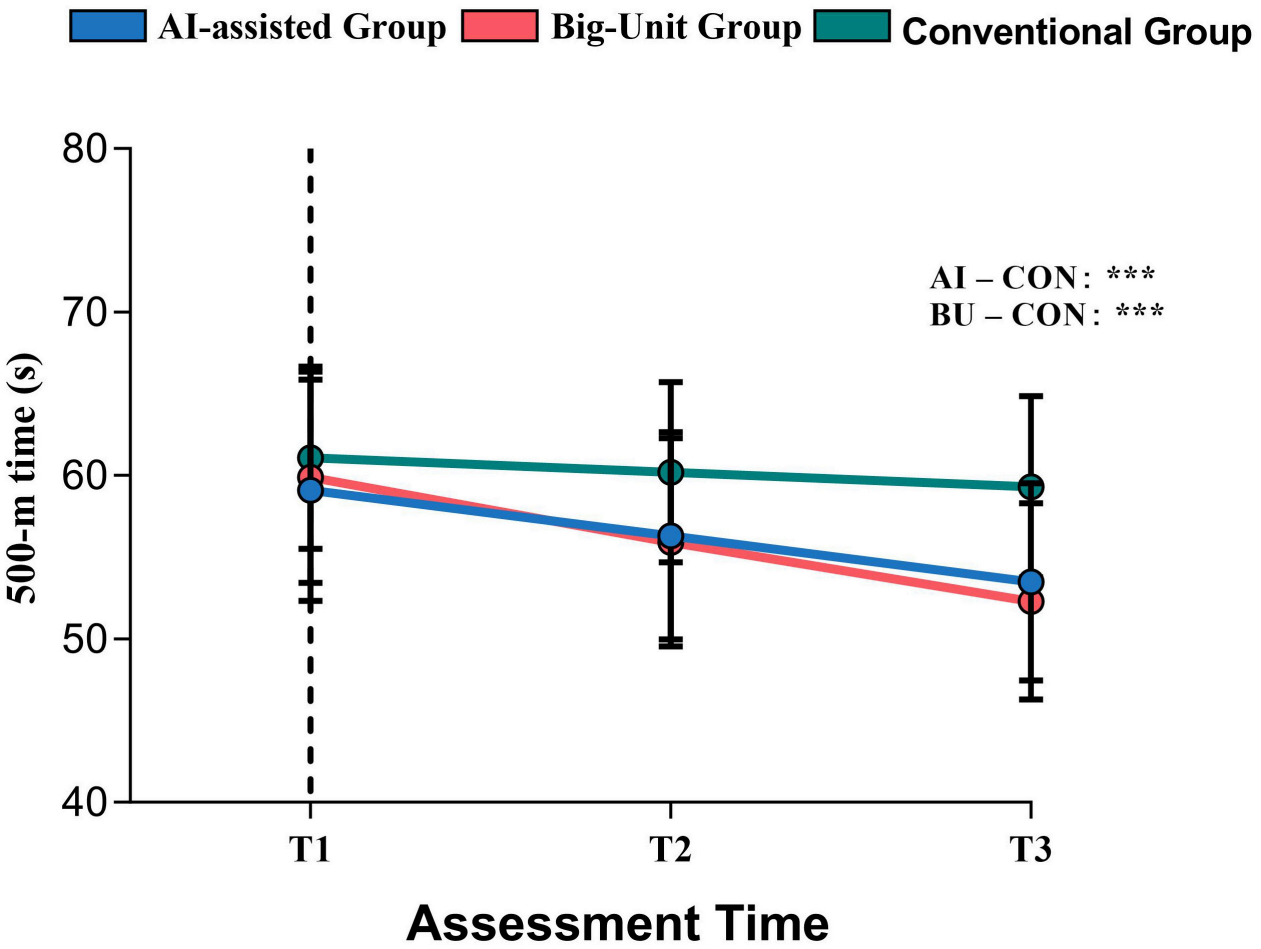
Longitudinal changes in 500-m speed-skating performance across T1–T3 by instructional group. Mean 500-m times (±SD) are shown for the AI-assisted group (*n* = 42), Big-Unit group (*n* = 43), and Conventional group (*n* = 44) at baseline (T1), mid-intervention (T2), and post-intervention (T3). Lower values indicate faster skating performance. Both AI-assisted and Big-Unit instruction produced substantially greater performance improvements than Conventional teaching, consistent with the significant Group × Time interaction from the longitudinal mixed-effects model [χ^2^_(4)_ = 364.18, *p* < 0.001]. Asterisks indicate considerable between-group contrasts at T3: ***p < 0.001 for AI vs. Conventional and Big-Unit vs. Conventional. For transparency, Big-Unit also showed a numerically faster post-test mean than AI-assisted (52.3 vs. 53.5 s; 1.2 s difference), which is treated as descriptive in the Results and discussed in Section 4.2.1.

As illustrated in Figure 3, both AI-assisted and Big-Unit teaching produced substantial reductions in 500-m time across T1–T3. By T3, mean times had decreased to 53.5 ± 6.04 s in the AI group and 52.3 ± 6.00 s in the Big-Unit group, corresponding to mean changes of −5.59 ± 1.10 s and −7.60 ± 1.86 s from baseline, respectively. In contrast, the Conventional group showed only modest improvement (T3: 59.3 ± 5.56 s; Δ = −1.80 ± 0.82 s). Within-group repeated-measures Cohen's d indicated substantial pre–post effects for all groups but particularly for AI and Big-Unit instruction (AI: |*d*| = 5.10; Big-Unit: |*d*| = 4.09; Conventional: |*d*| = 2.20).

Importantly, the Big-Unit group showed a numerically larger pre–post improvement than the AI-assisted group (ΔT3 – T1: −7.60 ± 1.86 s vs. −5.59 ± 1.10 s), corresponding to an approximate 1.2 s difference at post-test (52.3 vs. 53.5 s). These exceptionally large effect sizes (|*d*| > 4.0) reflect the sensitive nature of timing-based performance in elite-led instructional contexts. Because the primary inferential model focused on between-mode differences relative to Conventional instruction, this AI-vs.-Big-Unit contrast is presented here as a descriptive comparison and is discussed in the Discussion (Section 4.2.1).

The primary longitudinal mixed-effects model (LMM) for 500-m time, adjusting for age, gender, and BMI, revealed highly significant main effects of Time and Group, as well as a robust Group × Time interaction. Adding Time to a model without Time resulted in a substantial improvement in fit [likelihood-ratio test (LRT): χ^2^_(2)_ = 369.54, *p* < 0.001], indicating substantial overall performance gains across T1–T3. The Group main effect averaged across time points was also significant [χ^2^_(2)_ = 17.57, *p* < 0.001]. Crucially, including the Group × Time interaction significantly improved model fit relative to a Group + Time model [χ^2^_(4)_ = 364.18, *p* < 0.001], indicating that the magnitude of performance improvement differed systematically across teaching modes.

Together with the descriptive changes (Table 2, Figure 3), these results demonstrate that AI-assisted and Big-Unit teaching yielded substantially larger performance gains than conventional instruction.

### 3.3 Psychological and learning outcomes

Longitudinal trajectories for all psychological and learning outcomes demonstrated pronounced Group × Time effects (Table 3). Thirteen constructs were examined, including primary and supplementary learning motivation, self-efficacy (main and supplementary scales), autonomous learning ability, cooperation competence, psychological resilience, emotional regulation, self-control, core competence, pleasure/enjoyment, exercise interest, and anxiety & stress.

**Table 3:** Longitudinal mixed-effects models: Group × Time interaction tests.

**Outcome**	**χ^2^**	***df***	***p*-value (LRT)**
Cooperation competence	519.50	4	<0.001
Learning motivation (supp.)	514.67	4	<0.001
Autonomous learning ability	506.52	4	<0.001
Self-efficacy (main)	504.18	4	<0.001
Emotional regulation	484.42	4	<0.001
Pleasure/enjoyment	474.06	4	<0.001
Learning motivation (primary)	472.09	4	<0.001
Exercise interest	419.72	4	<0.001
Psychological resilience	419.05	4	<0.001
Core competence	403.69	4	<0.001
Anxiety & stress	401.62	4	<0.001
Self-control	392.89	4	<0.001
Self-efficacy (supp.)	137.28	4	<0.001

All interaction tests remained significant after Benjamini–Hochberg FDR correction (q < 0.001). Models were estimated without covariates to preserve raw developmental trajectories.

**Figure 4:**
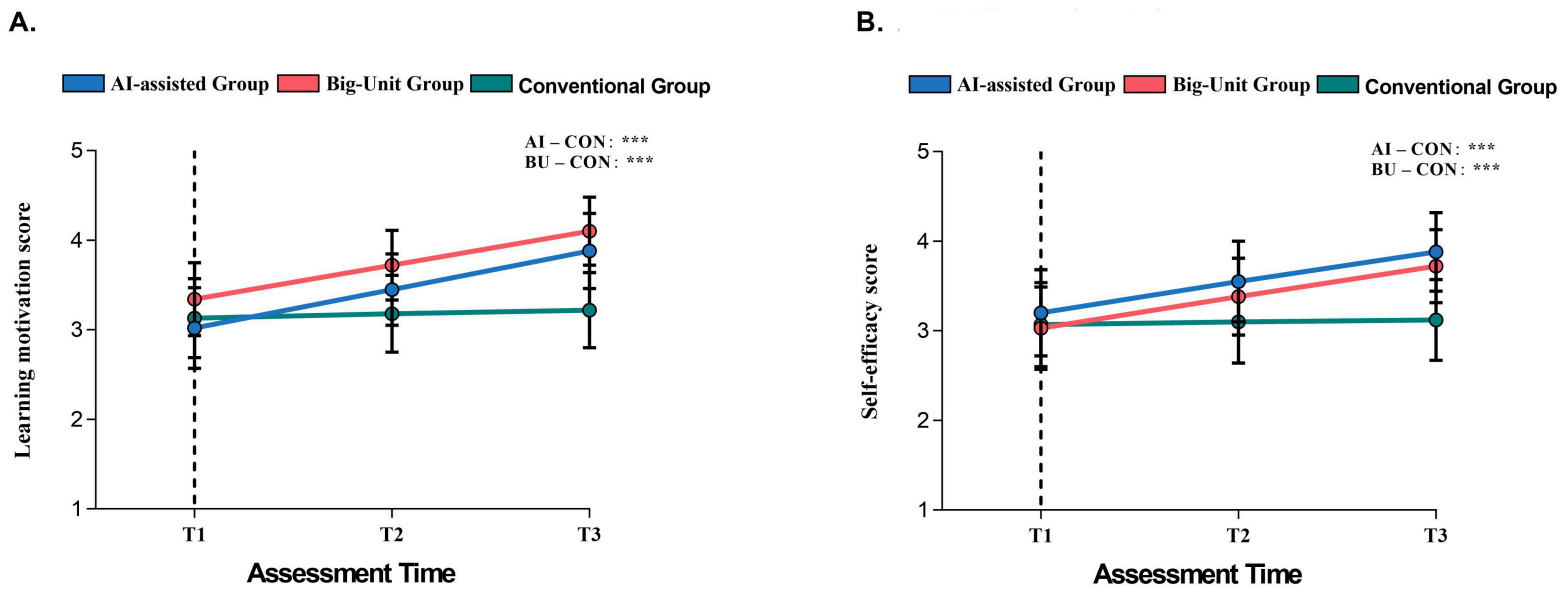
Longitudinal trajectories of motivation and self-efficacy across T1–T3. **(A)** shows changes in primary learning motivation, and **(B)** shows changes in self-efficacy (main scale) for the AI-assisted (*n* = 42), Big-Unit (*n* = 43), and Conventional (*n* = 44) groups across baseline (T1), mid-intervention (T2), and post-intervention (T3). Both innovative teaching modes produced substantially greater increases in motivation and self-efficacy relative to Conventional instruction, consistent with the significant Group × Time interaction effects reported in the longitudinal mixed-effects models [all χ^2^_(4)_ > 137.28, all *p* < 0.001]. Error bars represent ±1 SD. Asterisks indicate significant between-group contrasts at T3: ***p < 0.001 for AI vs. Conventional and Big-Unit vs. Conventional.

In all outcomes, LMMs with random participant intercepts showed significant Group × Time interactions [all χ^2^_(4)_ > 137.28, all *p* < 0.001; Table 3]. As illustrated in Figure 4, students in the AI-assisted and Big-Unit groups showed markedly steeper improvements across T1–T3, whereas those in the Conventional group showed minimal improvement. Improvements were especially prominent in motivation, self-efficacy, cooperation competence, and psychological resilience, alongside pronounced reductions in anxiety and stress.

Together, Table 3 and Figure 4 provide converging evidence that innovative teaching modes produced substantially stronger motivational and socio-emotional development relative to conventional instruction.

### 3.4 Adjusted group differences in change scores

To quantify adjusted group differences in pre–post change, ANCOVA models were conducted on change scores (Δ = T3 – T1) for five key outcomes: 500-m performance, learning motivation (supplementary), self-efficacy (supplementary), psychological resilience, and anxiety & stress (Table 4). Each model included Group as the primary predictor and adjusted for the baseline value of the corresponding outcome, age, gender, and BMI.

**Table 4:** ANCOVA results for change scores (Δ = T3 – T1).

**Outcome**	***F*_(2, 122)_**	***p*-value**	**Partial η^2^**
ΔSkillTime	283.77	<0.001	0.819
ΔLearningMotivation (supp.)	647.21	<0.001	0.927
ΔSelfEfficacy (supp.)	103.02	<0.001	0.650
ΔPsychologicalResilience	493.42	<0.001	0.902
ΔAnxietyStress	434.17	<0.001	0.909

Models adjust for baseline values (T1), age, gender, and BMI.

As summarized in Table 4, all ANCOVA models revealed highly significant Group effects with huge effect sizes. For ΔSkillTime, the Group effect was *F*_(2, 122)_ = 283.77, *p* < 0.001, partial η^2^ = 0.819, demonstrating that both AI-assisted and Big-Unit instruction produced substantially greater performance improvements than the Conventional group. Psychological outcomes showed similarly large Group effects:

- ΔLearningMotivation (supp.): *F*_(2, 122)_ = 647.21, *p* < 0.001, partial η^2^ = 0.927
- ΔSelfEfficacy (supp.): *F*_(2, 122)_ = 103.02, *p* < 0.001, partial η^2^ = 0.650
- ΔPsychologicalResilience: *F*_(2, 122)_ = 493.42, *p* < 0.001, partial η^2^ = 0.902
- ΔAnxietyStress: *F*_(2, 122)_ = 434.17, *p* < 0.001, partial η^2^ = 0.909

These results indicate that, after adjustment for baseline differences and demographic covariates, innovative teaching modes yielded markedly larger gains in motivation, self-efficacy, and resilience, along with significantly greater reductions in anxiety and stress.

### 3.5 Associations and mediation: psychological mechanisms linking teaching mode to performance

#### 3.5.1 Associations between performance change and psychological changes

In this section, we explore the associations between changes in performance (ΔSkillTime = T3 – T1, where negative values indicate better/faster performance) and psychological variables (Table 5). Pearson correlation analyses using complete cases (*N* = 129) revealed strong associations between ΔSkillTime and changes in psychological factors. Specifically, improvements in learning motivation (ΔLM) were strongly linked to better performance (*r* = −0.659, *p* < 0.001). Similarly, increases in self-efficacy (ΔSE; *r* = −0.510, *p* < 0.001) and psychological resilience (ΔRES; *r* = −0.543, *p* < 0.001) were significantly associated with greater performance improvement. Conversely, increased anxiety and stress (ΔAS) were associated with smaller performance gains (*r* = 0.462, *p* < 0.001).

**Table 5:** Correlations between performance change and psychological changes (complete cases, *N* = 129).

**Psychological change variable**	***n***	***r* with ΔSkillTime**	***p***
Learning motivation (ΔLM)	129	−0.659	<0.001
Self-efficacy (ΔSE)	129	−0.510	<0.001
Psychological resilience (ΔRES)	129	−0.543	<0.001
Anxiety & stress (ΔAS)	129	0.462	<0.001

ΔSkillTime = SkillTime_T3 – SkillTime_T1; negative values indicate faster performance (greater improvement).

#### 3.5.2 Mediation via learning motivation (SUPP; ANCOVA-style)

In an ANCOVA-style mediation model, we examined whether post-test learning motivation statistically accounted for the association between innovative teaching (AI + Big-Unit vs. conventional) and post-test performance (SkillTime_T3; lower values indicate better performance). The mediator equation adjusted for baseline learning motivation (LM_supp_T1), baseline performance (SkillTime_T1), age, gender, and BMI; the outcome equation (primary model) adjusted for baseline performance (SkillTime_T1), age, gender, and BMI. Bootstrap percentile confidence intervals were obtained from 2,000 resamples (complete cases, *N* = 129).

Innovative teaching was associated with higher post-test learning motivation (LM_supp_T3; path *a* = 1.131, 95% CI [1.008, 1.254], *p* < 0.001). In the outcome equation, LM_supp_T3 was positively associated with SkillTime_T3 after covariate adjustment (path *b* = 2.479, 95% CI [0.532, 4.643], *p* = 0.026). Accordingly, the indirect effect was significant (*a* × *b* = 2.804, 95% CI [0.570, 5.427], *p* = 0.038), while the direct effect of innovative teaching remained strongly negative (*c*′ = −7.957, 95% CI [−10.985, −5.217], *p* < 0.001), indicating an inconsistent (suppression-type) statistical mediation pattern rather than a fully explanatory causal pathway. The total effect was also significant (total = −5.153, 95% CI [−5.718, −4.570], *p* < 0.001).

Crucially, because the performance outcome is measured in seconds (where lower values signify improvement), this positive indirect effect (*ab* = 2.804) indicates a statistical suppression-type mediation pattern rather than a simple complementary pathway. This suggests that while innovative instruction drastically reduced total skating time, the independent psychological variance captured by the mediator functioned as a suppressor in the full model. The total effect remained significant and strongly negative (total = −5.153, 95% CI [−5.718, −4.570], *p* < 0.001), representing an overall reduction in skating time.

A sensitivity model additionally including baseline learning motivation (LM_supp_T1) in the outcome equation yielded a comparable indirect effect (2.816, 95% CI [0.702, 5.384], *p* = 0.028), supporting the robustness of this statistical indirect association.

**Figure 5:**
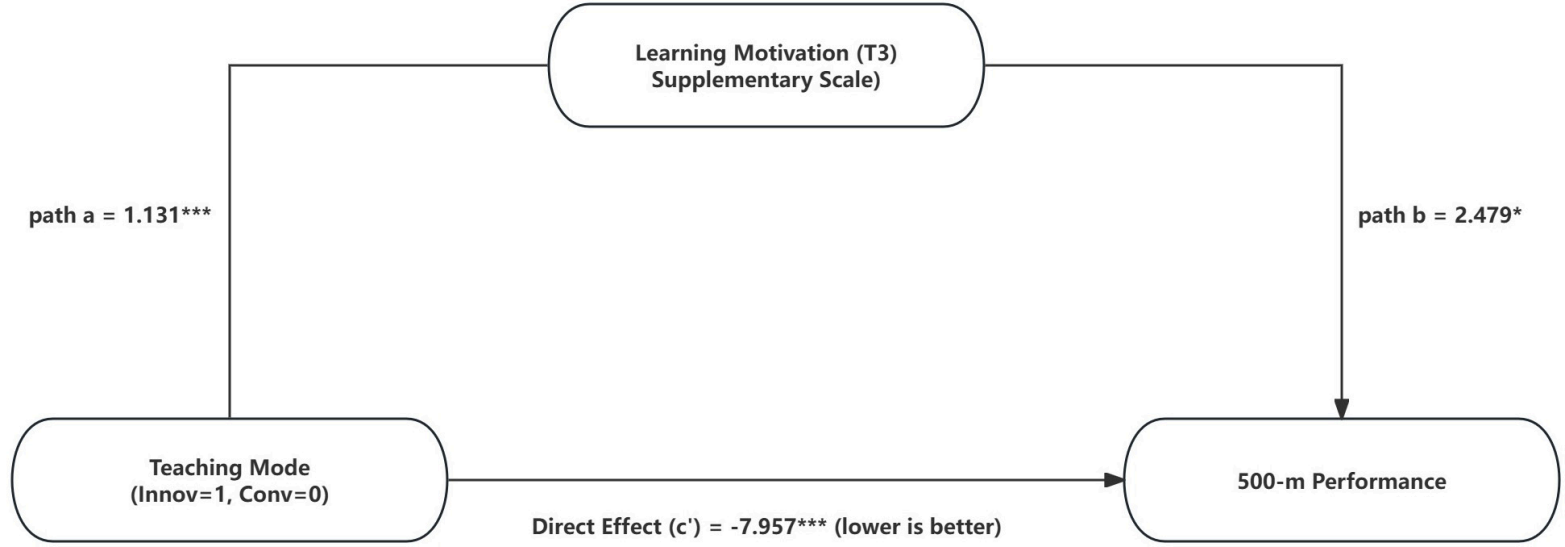
Mediation model linking teaching mode to 500-m speed-skating performance via learning motivation. All reported values are unstandardized coefficients (est). Paths were adjusted for covariates, including baseline (T1) scores, age, gender, and BMI. The total effect of innovative teaching was a significant reduction in skating time (Total = −5.153 s, *p* < 0.001). Crucially, since performance is measured in seconds, the positive indirect effect (*ab* = 2.804, 95% CI [0.570, 5.427]) represents the statistical reduction in time (improvement) accounted for by increased learning motivation. The direct effect (*c*′ = −7.957) remains the primary driver of performance gains. This illustrates a suppression-type mediation pattern, where the massive technical impact of instructional innovation is complemented, yet statistically distinct from, the motivational pathway.

As illustrated in Figure 5, the direct effect (*c*′ = −7.957) remains the primary driver of performance gains. This highlights a suppression-type framework where the technical impact of instructional innovation is the primary factor. Notably, both learning motivation and self-efficacy showed significant indirect effects within this statistical framework, mediating the relationship between innovative teaching and performance improvement.

#### 3.5.3 Mediation via self-efficacy (SUPP)

We next tested self-efficacy as the mediator using the same ANCOVA-style framework. The mediator equation adjusted for baseline self-efficacy (SE_supp_T1), baseline performance (SkillTime_T1), age, gender, and BMI; the outcome equation (primary model) adjusted for baseline performance (SkillTime_T1), age, gender, and BMI (complete cases, *N* = 129; bootstrap = 2,000).

Innovative teaching significantly increased post-test self-efficacy (SE_supp_T3; path *a* = 0.456, 95% CI [0.389, 0.525], *p* < 0.001). SE_supp_T3 was positively associated with SkillTime_T3 in the adjusted outcome equation (path *b* = 1.786, 95% CI [0.239, 3.394], *p* = 0.026), yielding a significant indirect effect (*a* × *b* = 0.814, 95% CI [0.109, 1.608], *p* = 0.030). The direct effect remained significant (*c*′ = −5.737, 95% CI [−6.793, −4.728], *p* < 0.001), with a significant total effect (total = −4.923, 95% CI [−5.389, −4.466], *p* < 0.001). Results were robust when baseline self-efficacy (SE_supp_T1) was additionally included in the outcome equation (indirect = 0.802, 95% CI [0.110, 1.558], *p* = 0.027).

### 3.6 Sensitivity analyses and robustness checks

A series of sensitivity analyses was conducted to evaluate the robustness of the primary findings. First, repeated-measures ANOVA (RM-ANOVA) was performed for 500-m performance, learning motivation (supplementary), self-efficacy (supplementary), and psychological resilience, specifying Time as the within-subject factor and Group as the between-subject factor (with the appropriate subject-level error term). Across all four outcomes, RM-ANOVA showed highly significant main effects of Time and Group, as well as Group × Time interactions (all *p* < 0.001), closely mirroring the linear mixed-model (LMM) results and indicating that conclusions were not contingent on the longitudinal modeling framework.

Second, we assessed the influence of covariate adjustment strategies on the Group × Time interaction tests in LMMs. For SkillTime, learning motivation (supplementary), self-efficacy (supplementary), and psychological resilience, the likelihood-ratio test (LRT) statistics for the interaction remained extremely large and highly significant under alternative specifications, including unadjusted models, models adjusted for age only, and fully adjusted models controlling for age, gender, and BMI [all χ^2^_(4)_ ≥ 137.28, all *p* < 0.001], supporting robustness to reasonable variations in covariate control.

Third, outlier robustness was examined by excluding participants with extreme change scores (|z| > 3) on any of the five key Δ variables. This procedure removed one participant (128/129 retained). Re-estimated LMMs and ANCOVAs in the reduced sample produced nearly identical patterns: the Group × Time interaction for SkillTime remained highly significant [χ^2^_(4)_ = 393.86, *p* < 0.001], and ANCOVAs on Δ outcomes continued to show strong Group effects (all *p* < 0.001), with effect sizes comparable to the primary analyses.

Finally, to address multiplicity across psychological outcomes, Benjamini–Hochberg false discovery rate (FDR) correction was applied to the LMM Group × Time interaction tests. All 13 outcomes remained significant after FDR adjustment (all *q* < 0.001), indicating that the observed pattern of psychological benefits under innovative teaching is unlikely to be attributable to chance.

Taken together, these sensitivity analyses consistently demonstrate that the performance and psychological advantages of AI-assisted and Big-Unit teaching over conventional instruction are statistically robust across analytic choices. Mediation results (Table 6) are interpreted as **statistical indirect associations** rather than definitive causal mechanisms, given the quasi-experimental intact-class design and the concurrent (post-test) measurement of mediators and performance.

**Table 6:** ANCOVA-style mediation analyses of innovative teaching on post-test performance via learning motivation and self-efficacy (complete-case *N* = 129; bootstrap = 2,000, percentile CI).

**Mediator**	***a* (95% CI)**	***p***	***b* (95% CI)**	***p***	**indirect *a* × *b* (95% CI)**	***p***	**Direct *c*^′^(95% CI)**	***p***	**Total (95% CI)**	***p***	***N***
**(A) Primary ANCOVA-style mediation models (recommended)**											
Learning motivation (LM_supp_T3)	1.131 [1.008, 1.254]	<0.001	2.479 [0.532, 4.643]	0.026	2.804 [0.570, 5.427]	0.038	−7.957 [−10.985, −5.217]	<0.001	−5.153 [−5.718, −4.570]	<0.001	129
Self-efficacy (SE_supp_T3)	0.456 [0.389, 0.525]	<0.001	1.786 [0.239, 3.394]	0.026	0.814 [0.109, 1.608]	0.030	−5.737 [−6.793, −4.728]	<0.001	−4.923 [−5.389, −4.466]	<0.001	129
**(B) Sensitivity models (add Mediator_T1 in outcome equation)**											
Learning motivation (LM_supp_T3)	1.131 [1.008, 1.254]	<0.001	2.490 [0.648, 4.582]	0.018	2.816 [0.702, 5.384]	0.028	−7.956 [−11.056, −5.236]	<0.001	−5.141 [−5.831, −4.448]	<0.001	129
Self-efficacy (SE_supp_T3)	0.456 [0.389, 0.525]	<0.001	1.759 [0.244, 3.280]	0.022	0.802 [0.110, 1.558]	0.027	−5.717 [−6.690, −4.722]	<0.001	−4.915 [−5.366, −4.458]	<0.001	129

Outcome: SkillTime_T3 (lower is better).

a: Mode_Innov → Mediator_T3; b: Mediator_T3 → SkillTime_T3; c′: direct effect of Mode_Innov on SkillTime_T3; indirect: a × b; total: c′ + a × b.

(A) (Primary): outcome equation controls SkillTime_T1 + Age + Gender + BMI; mediator equation controls Mediator_T1 + SkillTime_T1 + Age + Gender + BMI.

(B) (Sensitivity): same as (A) plus Mediator_T1 in the outcome equation.

The sensitivity model changes only the outcome equation by additionally adjusting for Mediator_T1; therefore, estimates for path a remain identical to the primary model.

## 4 Discussion

### 4.1 Summary of key findings

This study examined the effects of three teaching methods—AI-assisted teaching (AI group), Big-Unit teaching (Big-Unit group), and conventional teaching (Conventional group)—on 500-m speed-skating performance and psychological outcomes in first-year middle school students. Overall, the results demonstrate that both AI-assisted and Big-Unit teaching produced substantially greater improvements in performance and psychological functioning than conventional instruction (Sarkar and Fletcher, 2014; Jaitner et al., 2019; Hsia et al., 2025).

In terms of 500-m skating performance, both innovative teaching groups showed marked gains across the 8-week intervention. The AI group reduced their mean skating time from 59.1 ± 6.8 s at T1 to 53.5 ± 6.0 s at T3 (Δ = −5.59 ± 1.10 s), and the Big-Unit group improved from 59.9 ± 6.5 s to 52.3 ± 6.0 s (Δ = −7.60 ± 1.86 s). In contrast, the Conventional group exhibited only a modest reduction from 61.1 ± 5.6 s to 59.3 ± 5.6 s (Δ = −1.80 ± 0.82 s). ANCOVA on change scores, adjusting for baseline performance, age, gender, and BMI, confirmed a huge Group effect on ΔSkillTime [*F*_(2, 122)_ = 283.77, *p* < 0.001, partial η^2^ = 0.819; Table 4], supporting the conclusion that AI-assisted and Big-Unit teaching substantially outperformed conventional instruction in enhancing 500-m performance (Wulf and Lewthwaite, 2016).

Beyond performance, both innovative groups also showed pronounced improvements in psychological and learning-related variables. Students in the AI and Big-Unit groups experienced significant increases in learning motivation, self-efficacy, autonomous learning ability, cooperation competence, emotional regulation, and psychological resilience, alongside marked reductions in anxiety and stress over T1–T3. In contrast, students in the Conventional group exhibited only modest changes on these measures. Longitudinal mixed-effects models revealed robust Group × Time interactions for all 13 psychological outcomes [all χ^2^_(4)_ ≥ 137.28, all *p* < 0.001, FDR-adjusted *q* < 0.001; Table 3], and ANCOVA on key change scores (Δ learning motivation, Δ self-efficacy, Δ psychological resilience, Δ anxiety & stress) yielded substantial adjusted Group effects (partial η^2^ = 0.650–0.927; Table 4). Together, these findings indicate that the benefits of innovative teaching extended well beyond motor performance to encompass broad motivational and socio-emotional gains.

Mediation analyses were conducted as statistical decompositions of associations rather than tests of causal mechanisms, given the intact-class quasi-experimental design and the non-manipulated mediators. In the change-score (Δ) SEMs, teaching mode was associated with larger changes in learning motivation, and the indirect pathway via Δ learning motivation reached statistical significance (Table 6; Figure 5). However, the Δ-model displayed an inconsistent (suppression-type) pattern under covariate adjustment, such that the indirect effect carried an opposite sign to the total effect; accordingly, these results are interpreted as a partial statistical indirect effect (plausible pathway) rather than evidence of a causal mechanism. By contrast, the indirect effect via Δ self-efficacy did not reach statistical significance in the change-score models. T3 endpoint models that adjusted for baseline levels are treated as supplementary robustness checks indicating that post-test motivation and self-efficacy co-vary with the advantages of innovative instruction, rather than constituting primary mechanistic evidence.

Psychological resilience also emerged as an essential outcome closely linked to performance. The substantial gains in resilience observed in the AI and Big-Unit groups suggest that these teaching approaches help build students' capacity to cope with challenge and persist under pressure. The interactive, data-informed feedback in AI-assisted teaching and the progressive, mastery-oriented task structure in Big-Unit teaching likely provided repeated opportunities to experience manageable difficulty, recover from errors, and consolidate confidence (Sarkar and Fletcher, 2014).

Overall, the findings underscore that AI-assisted and Big-Unit teaching methods confer dual benefits: they markedly enhance 500-m skating performance while simultaneously strengthening key psychological resources, including learning motivation, self-efficacy, and resilience. These results contribute to the literature on educational psychology and sport pedagogy and offer practical guidance to educators seeking to integrate technology-enhanced, mastery-oriented approaches into physical education. By jointly fostering physical and psychological development, such teaching modes provide a holistic pathway to improving both athletic and mental health outcomes in adolescent learners.

### 4.2 Discussion of results

This study provides convergent evidence that AI-assisted and Big-Unit teaching methods are highly effective in improving both athletic performance and psychological wellbeing among middle school students in a winter-sport PE context. In this section, we interpret these findings through relevant educational and motivational theories and consider their implications for physical education practice.

#### 4.2.1 Teaching modes and athletic performance

The significant improvements in 500-m skating performance observed in the AI and Big-Unit groups highlight the potency of these instructional approaches for accelerating motor skill acquisition. Both groups achieved substantially greater reductions in skating time than the Conventional group, even after adjustment for baseline performance and demographic covariates. This pattern aligns with cognitive–behavioral and motor-learning perspectives, which emphasize the importance of structured practice, timely feedback, and progressive challenge for skill development (Paas et al., 2004; Roediger et al., 2011; Wulf and Lewthwaite, 2016). Specifically, the provision of external feedback and task progression has been shown to facilitate motor skill acquisition by enabling learners to focus on critical aspects of technique (Abbas and North, 2018).

Notably, the Big-Unit group achieved a numerically larger pre–post improvement than the AI-assisted group (approx. 1.2 s difference at T3). Although the study was not primarily powered for a formal superiority test between innovative modes, this contrast is pedagogically meaningful. Accordingly, this AI-vs.-Big-Unit difference is reported descriptively and should not be interpreted as evidence of superiority. A plausible explanation is that Big-Unit instruction provides a holistic and rhythm-focused skill integration through extended, coherent practice blocks. This “macro-level” scaffolding may be particularly advantageous for a 500-m task, where pacing strategy and rhythm stability are critical constraints on performance. In contrast, while the AI group benefited from precise “micro-level” kinematic feedback, the frequency and technical specificity of such data might impose a higher cognitive load or cause attentional fragmentation in some adolescent learners, potentially interfering with the automation of a fluid skating rhythm. This is consistent with Cognitive Load Theory (Roediger et al., 2011), which suggests that for novice-to-intermediate learners, an overabundance of external data can exceed working memory capacity, leading to “explicit monitoring” that hampers movement fluidity.

Two contextual features of this sample are essential for interpreting the magnitude of these effects and the unusually large effect sizes observed (e.g., partial η^2^ = 0.819 for ΔSkillTime). First, all participating students had engaged in compulsory speed-skating classes each winter since primary school. As a result, they entered the intervention with basic but non-professional competence—able to skate independently, control posture to some extent, and complete 500-m trials—but without systematic technical training, data-informed feedback, or mastery-oriented progression. This “intermediate” profile meant that their technique contained substantial inefficiencies that could be rapidly corrected through “technical renovation.” In technically demanding sports like speed skating, correcting foundational errors often leads to “discontinuous” leaps in performance, which explains the high Cohen's d values compared to more general physical activities.

Second, speed skating in this region is a seasonally constrained sport. Under conventional teaching, this short seasonal opportunity is only partially exploited through repetitive drills. In contrast, the present intervention maximized this window by reorganizing the same seasonal exposure into a tightly structured programme. The large between-group contrasts likely reflect a “limited headroom under conventional instruction” in the Conventional group, where lack of individualized feedback led to stagnation. In contrast, the innovative modes facilitated what Ericsson defines as deliberate practice, providing the necessary corrective cues to bypass early-career technical plateaus typical in high-complexity sports (Ericsson et al., 2018).

Within this context, the mechanisms of the two innovative approaches are complementary. In AI-assisted teaching, data-driven feedback supports rapid error detection and correction. The motion-recognition system provides precise information about stride parameters, posture, and trajectory, which the teacher translates into concrete, competence-supportive cues. This combination of objective analytics and pedagogical scaffolding likely enhances error awareness, refines movement patterns, and strengthens perceptions of competence, thereby enabling faster performance improvements.

By contrast, Big-Unit teaching operates primarily through curriculum structure rather than technology. By organizing content into coherent, extended units with clear mastery criteria, Big-Unit instruction enables students to build complex skills through stepwise progression. Early units focus on foundational control and safety, while later stages incorporate more complex skills such as pacing and rhythm stability, which are critical for performance in a 500-m event (Carpintero et al., 2009). This structure not only reduces cognitive overload but also promotes sustained engagement by allowing students to experience mastery and progress at each stage. The present results indicate that such a mastery-oriented progression can be at least as effective as AI-assisted feedback in improving 500-m performance, suggesting that structural curriculum design and technology-enhanced feedback represent complementary rather than mutually exclusive pathways to performance enhancement. At the same time, the seasonal and prior-experience context implies that effects may differ in true beginners or in non-seasonal sports, an issue future studies should address explicitly.

#### 4.2.2 Psychological variables: learning motivation and resilience

The pronounced improvements in learning motivation and psychological resilience in the AI and Big-Unit groups are consistent with Self-Determination Theory (SDT) and contemporary resilience frameworks (Venâncio et al., 2025). SDT posits that motivation is optimized when learners experience autonomy, competence, and relatedness (Humphry, 2013). In the AI condition, real-time feedback—mediated by the teacher—likely enhanced feelings of competence by making progress visible and providing actionable guidance. In contrast, teacher regulation of pacing and interpretation of feedback helped maintain autonomy and relatedness. In the Big-Unit condition, progressive task design and explicit mastery criteria enabled students to experience repeated cycles of challenge and success, thereby reinforcing competence and supporting self-determined forms of motivation.

Psychological resilience, conceptualized as adaptive functioning under challenge, also improved markedly under innovative instruction. Both AI-assisted feedback and Big-Unit progression expose students to structured difficulty: they repeatedly confront demanding tasks (e.g., higher speeds, tighter pacing constraints), receive feedback, adjust their strategies, and eventually succeed. This process aligns with cognitive–behavioral accounts in which successful coping experiences and mastery of challenging tasks reinforce self-efficacy, promote adaptive appraisals of stressors, and foster greater persistence (Sarkar and Fletcher, 2014). The observed reductions in anxiety and stress, particularly in the AI and Big-Unit groups, suggest that students not only became more skilled but also developed greater confidence and emotional regulation when performing in a potentially threatening ice environment.

#### 4.2.3 Mediation of learning motivation

The mediation analyses were conducted to examine plausible statistical pathways (i.e., decompositions of associations) rather than to demonstrate causal mechanisms, given the intact-class quasi-experimental allocation and the fact that mediators were not experimentally manipulated. In the change-score (Δ) models, innovative teaching was associated with larger gains in learning motivation (path a; Table 6), and the indirect pathway via Δ learning motivation reached statistical significance (Table 6). However, the Δ-model also showed an inconsistent (suppression-type) pattern under covariate adjustment, in which the indirect effect carried an opposite sign to the total effect.

##### 4.2.3.1 Cognitive load and technical dominance explanation

In practical terms, this suppression-type effect indicates that after accounting for the strong direct effect of teaching mode and covariates, the residual variance in motivation change was inversely associated with the improvement index, producing an indirect effect that did not align with the direction of the overall teaching advantage. In the Δ-model, the positive indirect effect (*ab* = 2.804) indicates that for every unit of motivational gain, there was a corresponding statistical reduction in skating time (i.e., “seconds saved”). The suppression pattern arises because the intervention's direct impact on technical speed far exceeded the variance accounted for by this motivational “savings” channel.

One possible explanation for this phenomenon lies in cognitive load theory (Roediger et al., 2011), which suggests that learners can become overwhelmed if the amount of feedback exceeds their cognitive processing capacity. In the context of AI-assisted teaching, students may have been highly motivated initially by the system's precise, real-time feedback. However, this overabundance of data may have led to attentional fragmentation, where students focused too much on isolated details (such as posture or stride mechanics) rather than integrating these movements into a fluid skating rhythm. This aligns with Sweller's cognitive load theory, which posits that excessive external information can overwhelm working memory and impede the learner's ability to automate movements efficiently (Roediger et al., 2011). As a result, the increased motivation observed in the AI group did not always translate to faster performance, suggesting that motivational boosts could have interfered with performance optimization.

By contrast, the direct effect of teaching mode in the Big-Unit group remained strongly negative (path *c*′ = −7.957, *p* < 0.001), which highlights the dominant role of the technical instructional component in driving performance gains. This suggests that, despite the initial motivational boost in both innovative groups, the structure of the Big-Unit teaching (progressive mastery and clear criteria) ultimately had the greatest impact on performance. Technical renovation, a term used in the literature on high-skill sports, refers to correcting foundational errors that lead to disproportionate performance gains (Ericsson et al., 2018). In speed skating, where technique is highly technical and crucial for improvement, the rapid correction of such errors under a structured teaching method may have yielded discontinuous improvements, which accounts for the large effect size observed in the Big-Unit group.

Therefore, while the Δ-model findings should not be interpreted as evidence that increased motivation causally produced faster skating, they offer a nuanced explanation of how motivational change covaries with performance change in this dataset. The suppression effect suggests that the increased motivation observed in the AI group did not directly drive better performance. Still, rather, both motivation and performance were influenced by the technical and cognitive aspects of the teaching methods, particularly the “technical renovation” that occurred within the Big-Unit method.

##### 4.2.3.2 Matthew effect: baseline differences and self-criticism

Notably, baseline differences in psychological variables, particularly learning motivation, might have contributed to the outcomes. While we statistically controlled for baseline values, we cannot rule out the possibility that students with higher initial motivation might have been more receptive to the autonomous nature of the Big-Unit structure, potentially amplifying the observed effects. This creates a Matthew effect (Merton, 1968), where students with a higher baseline level of motivation benefited disproportionately from the intervention. This is a standard limitation in quasi-experimental designs and could have contributed to the larger gains observed in the Big-Unit group. While we controlled for baseline levels in our statistical models, we acknowledge that the initial differences in motivation may have created an inherent bias in the analysis. This self-criticism recognizes that the group with higher initial motivation may have been more receptive to the Big-Unit approach, which requires students to self-regulate and master skills progressively.

##### 4.2.3.3 Indirect pathway via Δ self-efficacy

In the same change-score framework, the indirect pathway via Δ self-efficacy did not reach statistical significance (Table 6), suggesting that although efficacy gains co-occur with performance improvements, they did not form a statistically robust indirect channel in the Δ models. Endpoint (T3) mediation models that adjust for baseline levels are reported as supplementary robustness checks, indicating that post-test motivation and self-efficacy states align with instructional advantages even when change-score pathways exhibit suppression. However, the absence of a significant indirect effect via self-efficacy may reflect that changes in self-efficacy were less predictive of performance changes than changes in learning motivation, aligning with other studies that show motivation as a stronger driver of performance in physically demanding tasks (Ryan and Deci, 2000).

##### 4.2.3.4 Theoretical perspective on motivation and mastery-oriented pedagogy

From a theoretical perspective, the broader pattern—strong improvements in motivational climate under innovative teaching alongside large performance gains—is consistent with autonomy-supportive and mastery-oriented pedagogy, which can enhance engagement quality, persistence, and willingness to invest effort in technically demanding tasks (Ryan and Deci, 2000). In the present context, both AI-assisted feedback (when delivered in a competence-supportive manner) and Big-Unit mastery progression likely created learning environments in which challenges were framed as manageable and improvable, thereby supporting sustained participation and practice quality. Nevertheless, given the quasi-experimental design and the suppression-type result in Δ mediation, these interpretations are offered as theoretically grounded explanations rather than confirmed causal mechanisms.

#### 4.2.4 Implications for educational practice

Several practical implications emerge from these findings. First, the success of AI-assisted and Big-Unit teaching underscores the value of designing PE environments that are both informationally rich and structurally coherent. AI-based tools can help teachers deliver individualized, timely feedback that may be difficult to provide through observation alone, particularly in large classes or technically demanding activities such as speed skating. When used ethically and under teacher supervision, such tools can enhance, rather than replace, pedagogical judgment by providing precise, actionable data to guide instruction.

Second, Big-Unit teaching demonstrates that substantial gains in both performance and psychological functioning can be achieved through careful task sequencing and explicit mastery criteria, even without advanced technology. This is particularly relevant for schools with limited access to digital resources. By breaking complex skills into manageable components and allowing sufficient time for consolidation within each unit, Big-Unit teaching can support students with diverse ability levels and promote a sense of progress and competence over time.

Third, the strong motivational and resilience effects observed here suggest that PE curricula should explicitly target psychological development alongside physical skill outcomes. Integrating elements such as goal setting, reflective practice, peer support, and discussion of coping strategies into AI-assisted and Big-Unit frameworks may further strengthen motivation and resilience. Such an approach aligns with broader educational aims of fostering lifelong engagement in physical activity and equipping students with psychological tools to manage stress and challenge in both sport and everyday life.

In sum, the present findings indicate that PE teachers can leverage both technology-driven feedback and mastery-oriented curriculum structure to create learning environments that are physically demanding, psychologically supportive, and developmentally appropriate. Thoughtful integration of these elements has the potential to improve not only students' sport-specific performance but also their motivation, confidence, and resilience in the face of challenge.

### 4.3 Implications for future research

The findings from this study offer significant insights into the effectiveness of AI-assisted and Big-Unit teaching methods in physical education. While this research provides a solid foundation, several critical areas remain for future exploration to enhance further and refine the application of these methods. Below, we outline key directions for future research based on the present findings and their practical implications.

#### 4.3.1 Long-term effects and application across age groups

A key avenue for future research is to examine the long-term effects of AI-assisted and Big-Unit teaching methods on students' athletic performance and psychological wellbeing. This study focused on a relatively short-term intervention, and the long-term sustainability of the observed effects remains unknown. Longitudinal studies would enable researchers to track how these teaching methods influence students as they progress through different stages of schooling.

In addition, future research should assess the effects of these methods across a wider age range. Younger students or those with limited prior experience may require more scaffolded support—such as more structured feedback or gradual task progression. In contrast, older or more experienced students might benefit from greater autonomy and less direct supervision. Understanding how age and prior experience influence the efficacy of AI-assisted and Big-Unit teaching methods will be crucial for tailoring interventions to specific developmental stages and educational levels (Telama, 2009).

#### 4.3.2 Individual differences and personalized interventions

Research on individual differences—such as gender, prior athletic experience, baseline motivation, or psychological profile—is another important direction for future work. These factors may moderate the effectiveness of AI-assisted and Big-Unit teaching methods. For example, students with greater prior athletic experience may require less scaffolding and may show different rates or patterns of improvement than those with less experience. Gender differences may also influence how feedback is perceived and acted upon, with male and female students potentially responding differently to AI-driven feedback or progressively challenging tasks.

Future studies should investigate how to adapt these teaching methods to meet students' diverse needs by tailoring interventions to their individual characteristics, thereby ensuring a more personalized learning experience (Wadsworth et al., 2013; Allen and Laborde, 2014). Such adaptations could involve adjusting the intensity, frequency, and specificity of feedback or calibrating task difficulty according to students' initial skill levels, psychological readiness, and personal goals (Wadsworth et al., 2013).

#### 4.3.3 Ecological validity and real-world implementation

Although the present study was conducted in an authentic school-based winter-sport context, it was limited to a single school in a specific regional setting. To strengthen ecological validity and real-world applicability, future research should examine how AI-assisted and Big-Unit teaching methods perform under more varied and dynamic educational conditions. In everyday PE practice, factors such as school resources, teacher training, class size, and timetable constraints often differ substantially from those in a focused research implementation.

For instance, AI-assisted teaching tools could be tested in schools with limited access to technology to explore how conditions in low-resource settings affect their feasibility and effectiveness. Similarly, researchers could examine how teachers in underfunded schools adapt Big-Unit teaching while maintaining coherent task progression under constraints in space, equipment, or instructional time. Such investigations would provide practical guidelines for scaling these interventions to schools with different resource profiles, helping to ensure that AI-assisted and Big-Unit methods are accessible and applicable across a wide range of educational environments (Doe-Asinyo and Smits-Engelsman, 2021; Kiyani et al., 2022).

#### 4.3.4 Integrating technology with traditional methods

Another promising direction is integrating AI-based teaching tools with traditional pedagogical approaches. While AI-assisted and Big-Unit teaching methods demonstrated positive effects when implemented as distinct conditions in this study, combining technology with established teaching practices may yield additional benefits. Future research should examine how real-time AI feedback can be effectively integrated with teacher–student interactions to create a more dynamic and individualized learning environment.

For example, AI systems could provide instant performance data, while teachers use their pedagogical expertise to interpret this data, offer individualized guidance, and foster supportive motivational climates. This blended approach has the potential to enhance the learning process by combining the precision of technology with the relational and contextual sensitivity of human instruction. Research on the synergy between AI-based tools and traditional methods could yield valuable insights into optimizing teaching strategies for maximum engagement and performance (Kiyani et al., 2022).

#### 4.3.5 Expanding the generalizability of findings

The current study focused on a specific cohort of first-year middle school students in a single regional context, limiting the generalizability of the findings. Future research should investigate how AI-assisted and Big-Unit teaching methods can be applied across different age groups, sports disciplines, and cultural contexts. For instance, it remains unclear whether similar benefits would emerge in high school populations or in team sports such as basketball, football, or volleyball, where tactical coordination and interpersonal dynamics play a larger role.

Cross-cultural studies would also be valuable for understanding how cultural factors—such as attitudes toward technology, competition, and physical education—shape the effectiveness of these instructional methods. A more diverse set of samples would allow researchers to examine whether the observed effects are consistent across different demographic groups and educational systems, thereby enhancing the external validity of AI-assisted and Big-Unit teaching approaches (Jung et al., 2021; Jerebine et al., 2024).

#### 4.3.6 Methodological approaches and future directions

Finally, future studies could adopt more diverse methodological approaches to deepen understanding of how AI-assisted and Big-Unit teaching methods operate in practice. Qualitative methods—such as interviews with teachers, focus groups with students, or classroom observations—could provide rich, context-specific insights into the implementation challenges, perceived benefits, and day-to-day adaptations of these methods. Mixed-methods designs that integrate quantitative performance and psychological data with qualitative feedback would offer a more comprehensive picture of their impact on students' learning experiences.

Methodological approaches should also consider finer-grained longitudinal data to capture the trajectory of improvement and the influence of repeated exposure to AI feedback or Big-Unit training. Incorporating more granular indicators—for example, session-by-session performance metrics, real-time feedback logs, or micro-level engagement markers—could enable researchers to identify critical turning points in students' motivation, performance, and resilience (Miller et al., 2024; Ba et al., 2025).

### 4.4 Limitations and future research directions

While this study provides valuable evidence for the effectiveness of AI-assisted and Big-Unit teaching methods, several limitations must be acknowledged. These limitations help contextualize the findings and identify essential priorities for future research. Addressing these issues will strengthen understanding of how AI-assisted and Big-Unit instruction can be applied across diverse educational settings.

#### 4.4.1 Quasi-experimental design and causal inference

The primary limitation of this study lies in its intact-class, non-randomized allocation, which was necessitated by school administrative structures but limits definitive causal inference. Since students were not individually randomized, the teaching mode was inevitably confounded with class membership. This means that class-level factors—such as pre-existing peer dynamics, established teacher–student relationships, and specific classroom motivational climates—cannot be fully disentangled from the effects of the instructional interventions. As a result, the mediation findings reported in this study should be interpreted as plausible statistical pathways rather than confirmed causal mechanisms. Additionally, since one instructor delivered all sessions to ensure pedagogical consistency, the results may partially reflect teacher-specific factors, such as individual enthusiasm or anticipation of the success of the innovative methods.

To enhance internal validity, future research should transition to randomized controlled trial (RCT) designs, specifically cluster-randomized approaches involving multiple instructors across diverse school sites. Such designs would allow for a more accurate isolation of teaching-mode effects from class-level or contextual biases, providing a stronger foundation for evaluating the specific contributions of AI-assisted and Big-Unit instruction to student development (Rogers and Révész, 2019).

#### 4.4.2 Teacher-related factors and implementation bias

A significant factor influencing the efficacy of the interventions is the teacher's role. While employing a single instructor across all conditions ensured pedagogical consistency, this design choice also introduces potential implementation bias. Teacher-specific factors—including individual enthusiasm, professional experience, and teacher anticipation of the innovative methods' success—may have contributed to the observed results. Specifically, the “teacher expectancy effect” suggests that an instructor's belief in a new process can lead to more vigorous implementation and positive reinforcement, which, in turn, shape student outcomes (Rosenthal, 2002). Accordingly, the present findings may reflect not only the instructional models themselves but also the way this particular teacher enacted them.

This concern is particularly pertinent to AI-assisted teaching, where the instructor must interpret complex system outputs and translate them into supportive, actionable cues. A teacher's pro-technology stance might result in more frequent or more detailed feedback, inadvertently amplifying students' perceived competence and engagement. Likewise, the success of Big-Unit instruction relies heavily on the teacher's ability to operationalize mastery criteria, pace progression, and sustain a mastery-oriented climate. Because innovative conditions may naturally elicit greater teacher attention and novelty-related enthusiasm, it is difficult to entirely rule out “attention/novelty effects” as a partial contributor to the between-group differences.

Future research should therefore employ multiple instructors and incorporate explicit fidelity monitoring to disentangle teaching-mode effects from individual teacher characteristics. For example, structured fidelity checklists (session-by-session), third-party observations, and inter-rater agreement on adherence indicators (e.g., frequency/quality of AI feedback, implementation of mastery checks, and unit progression rules) would strengthen interpretability. In parallel, examining how teacher professional development can standardize delivery and reduce expectancy-driven variability will be essential for scaling these innovations across broader educational systems (Armour and Yelling, 2007; Darling-Hammond et al., 2017).

#### 4.4.3 Measurement bias and social desirability

Measurement bias remains a critical concern, as the study relied on self-reported psychological outcomes. Self-report instruments are inherently vulnerable to social desirability bias and limited self-awareness, where students may consciously or unconsciously inflate scores to present themselves favorably (Paulhus, 2012). This issue is especially relevant in school-based intervention studies because self-report outcomes can be affected by “common method variance,” which can inflate associations between perceived teaching quality, motivation, and psychosocial outcomes (Podsakoff et al., 2003).

In the present study, such bias may not be evenly distributed across groups. A novelty effect might have amplified the tendency toward socially desirable responding, as students in the AI-assisted and Big-Unit groups likely perceived their learning environment as “special” or “privileged.” Because these two conditions also involved more visible instructional attention (e.g., technology-supported feedback or explicit mastery structures), students may have inferred stronger expectations for “improvement,” which could systematically elevate post-test ratings of motivation, resilience, and enjoyment relative to the conventional group. Therefore, part of the observed between-group differences in psychological scales may reflect reporting tendencies rather than actual changes in latent constructs.

To mitigate these limitations, future research should employ methodological triangulation. Integrating self-reports with objective physiological markers—such as heart rate variability to assess stress regulation (Kim et al., 2018)—and systematic behavioral observations of on-task engagement would provide a more robust data profile. In addition, several low-burden design features could directly reduce social desirability and expectancy-driven responding: (1) administering questionnaires anonymously with explicit reassurance of no academic consequences, (2) using blinded or independent assessors when feasible, and (3) including brief social desirability or response-style checks to quantify and adjust for response bias (Paulhus, 1991). Finally, future studies should test measurement invariance across groups and time points to ensure that observed score changes reflect comparable constructs rather than shifts in scale interpretation under different teaching climates (Putnick and Bornstein, 2016).

#### 4.4.4 Sample size and generalizability

A key limitation of this study is the relatively modest sample size (*N* = 129), which may limit the generalizability of the findings to broader populations. Although the sample size was adequate to detect statistically significant effects, it may yield imprecise estimates of effect magnitude and may overstate effect sizes in a single-site quasi-experimental context. In particular, the extremely large effects observed here should be interpreted with caution, as they may reflect context-specific “ceiling/seasonal window” conditions rather than transportable impacts across typical PE settings (Gelman and Carlin, 2014).

Moreover, the sample was drawn from one school within a region with a strong winter-sport tradition and a distinctive cultural background, which may constrain external validity. Students' prior exposure to seasonal speed-skating classes, local facility access, and school-level emphasis on winter sports could have amplified responsiveness to both innovative interventions. Therefore, replication in other provinces, non-winter-sport schools, and settings with different baseline skill profiles is necessary before broader policy recommendations are made (Tipton, 2013).

Future research should employ larger, more heterogeneous samples to assess the wider applicability of these teaching methods. In particular, multi-site studies should include participants from diverse socioeconomic backgrounds and schools in both urban and rural regions, allowing examination of how AI-assisted and Big-Unit approaches perform across different educational environments (Cheung, 2019). Where feasible, cluster-randomized designs across multiple schools and teachers would strengthen both generalizability and causal interpretability, and would allow exploration of effect heterogeneity by baseline competence, resource availability, and cultural context (Hayes and Moulton, 2017).

#### 4.4.5 Ecological validity and real-world application

Although the intervention was implemented in an authentic school-based winter-sport context, ecological constraints may limit the scalability and transferability of the findings. The study was conducted in a single institution with relatively stable organizational support; however, real-world implementation of AI-assisted and Big-Unit instruction is likely to be shaped by variability in facility access, class size, teacher workload, and technological infrastructure. In many schools, limited availability of winter-sport venues, restricted access to timing systems or motion-recognition hardware, and uneven teacher readiness to adopt new models may reduce feasibility or attenuate effects.

A further ecological constraint specific to speed skating is seasonality. Instructional exposure is often compressed into a narrow winter window, which may inflate short-term gains when instructional time is concentrated, but may also limit the durability and generalizability of the effects across a full academic year. Under such seasonal conditions, intervention impact is partly contingent on the quantity and continuity of ice-time, which can be disrupted by weather conditions, facility scheduling, school events, or safety-related cancellations. Therefore, future implementation studies should treat ice-time availability and cancellation frequency as explicit fidelity indicators, documenting session completion rates and the stability of practice opportunities as part of intervention monitoring (e.g., fidelity checklists or third-party observations; Dusenbury et al., 2003; Carroll et al., 2007).

To improve scalability, future research should test hybrid implementation models that combine on-ice instruction with structured dry-land preparation (e.g., rhythm drills, balance and posture control, pacing simulations, and strength–coordination tasks). Such designs could extend learning opportunities beyond the winter window and reduce dependence on facility access while retaining core pedagogical principles. In parallel, “technology downscaling” strategies—such as mobile applications, simplified feedback dashboards, or offline AI models—may enable schools with limited resources to adopt a reduced-cost version of individualized feedback without requiring extensive hardware or continuous connectivity (Telama, 2009).

Furthermore, the success of such ecological adaptations—particularly the uptake and sustainability of structured Big-Unit progression—may be influenced by the school's organizational culture and the surrounding community's winter-sport traditions, factors that warrant specific consideration (Stalsberg and Pedersen, 2010).

#### 4.4.6 Long-term sustainability of gains

The relatively short duration of the intervention (eight weeks) limits inference regarding the sustainability and developmental trajectory of the observed gains. Although both innovative conditions produced substantial improvements in skating performance and psychological outcomes, it remains unclear whether these benefits would persist, plateau, or decay once the structured teaching inputs and seasonal practice opportunities are reduced. This issue is particularly salient for winter-sport PE contexts, where the end of the ice season may interrupt practice continuity and potentially weaken skill retention and motivational momentum.

Future research should therefore employ longitudinal follow-up designs (e.g., post-season and next-season assessments) to evaluate retention of technical improvements and the stability of psychological resources such as motivation, resilience, and self-efficacy. In addition, studies should test maintenance mechanisms that may consolidate gains, including periodic booster sessions, continued access to simplified feedback tools, and structured home-based or dry-land training that preserves rhythm and pacing competencies. Tracking students across transitions (e.g., grade progression, changes in class composition, or shifts to other sport units) would further clarify whether innovative teaching produces generalizable self-regulation benefits or primarily sport-specific adaptations. Establishing the durability of effects is essential for translating short-term intervention success into sustainable educational practice (Telama, 2009).

#### 4.4.7 Cultural specificity and generalizability

The cultural and regional context of the sample must be considered when interpreting the magnitude and transferability of the instructional effects. The participating school is situated in a community with a strong Korean heritage and a well-established tradition of winter sports. Such contexts may be characterized by collective discipline, high compliance with instructional norms, and strong peer-regulated practice cultures, which could amplify responsiveness to structured pedagogies. In particular, Big-Unit teaching relies on shared task progression, peer observation, and the internalization of mastery criteria; these features may be especially compatible with collectivistic learning norms and culturally reinforced respect for teacher authority. Therefore, part of the observed advantages—especially the strong motivational and socio-emotional gains—may reflect a context–intervention fit rather than a universally replicable magnitude of effect.

Moreover, the local familiarity with speed skating provided a distinctive “technical starting point.” Even without formal training, students likely entered the intervention with basic balance, posture control, and safety competence acquired through repeated seasonal exposure. This baseline “winter-sport literacy” may have enabled the innovative interventions to operate through rapid technical renovation (refinement of rhythm, pacing stability, and efficiency) rather than through elementary skill acquisition, thereby facilitating unusually large short-term gains. In regions without entrenched winter-sport traditions—or in school cultures where peer learning norms differ—the Big-Unit task chain and the uptake of AI feedback may yield different motivational dynamics and smaller effect sizes.

Future studies should test cross-cultural and cross-region replication using multi-site designs that explicitly compare schools with differing cultural compositions, organizational climates, and winter-sport exposure. Such work would clarify whether the current findings generalize to more individualistic learning cultures, non-winter sport PE settings, or student populations without prior skating experience, and would help identify which elements of AI-assisted and Big-Unit instruction are culturally robust vs. context-dependent (Stalsberg and Pedersen, 2010).

## 5 Conclusion

This study provides robust evidence that both AI-assisted teaching and Big-Unit teaching substantially enhance adolescent speed-skating performance and psychological functioning compared with conventional instruction. Across an 8-week intervention implemented in an authentic winter-sport PE environment, students receiving innovative instruction demonstrated superior gains in 500-m performance as well as meaningful improvements in learning motivation, self-efficacy, emotional regulation, cooperation competence, and psychological resilience. These findings reinforce the view that pedagogical innovation can support both motor-skill refinement and socio-emotional development—two core pillars of sustained engagement in technically demanding physical activities.

Mediation analyses identified learning motivation as the primary psychological mechanism underlying performance improvements, with self-efficacy contributing an additional but smaller pathway. These patterns align with motivational and resilience-based theories, underscoring the importance of competence-supportive, autonomy-supportive, and mastery-oriented learning environments in physical education.

Overall, the study offers new empirical evidence comparing micro-level (AI-assisted) and macro-level (Big-Unit) instructional approaches within the same winter-sport context and highlights their complementary potential for modernizing PE curricula. Future research should examine long-term effects, extend these approaches to other sports and developmental stages, and evaluate their feasibility in diverse school environments to enhance generalizability.

## Data Availability

The original contributions presented in the study are included in the article/supplementary material, further inquiries can be directed to the corresponding authors.
